# Recent Breakthroughs and Ongoing Limitations in *Cryptosporidium* Research

**DOI:** 10.12688/f1000research.15333.1

**Published:** 2018-09-03

**Authors:** Seema Bhalchandra, Daviel Cardenas, Honorine D. Ward

**Affiliations:** 1 Division of Geographic Medicine and Infectious Diseases, Tufts Medical Center, Boston, Massachusetts, 02111, USA; 2 Medicine, Public Health and Community Medicine, Tufts University School of Medicine, Boston, Massachusetts, 02111, USA

**Keywords:** Cryptosporidium, cell culture, genetic modification, transcriptomics, drug discovery

## Abstract

The intestinal apicomplexan parasite *Cryptosporidium* is a major cause of diarrheal disease in humans worldwide. However, treatment options are severely limited. The search for novel interventions is imperative, yet there are several challenges to drug development, including intractability of the parasite and limited technical tools to study it. This review addresses recent, exciting breakthroughs in this field, including novel cell culture models, strategies for genetic manipulation, transcriptomics, and promising new drug candidates. These advances will stimulate the ongoing quest to understand *Cryptosporidium* and the pathogenesis of cryptosporidiosis and to develop new approaches to combat this disease.

## Introduction

The intestinal apicomplexan parasite *Cryptosporidium* is responsible for waterborne outbreaks of diarrheal disease worldwide and continues to cause opportunistic infection in immunocompromised hosts, including patients with untreated HIV/AIDS ^1^. Recently, this parasite has been increasingly recognized as a major cause of diarrhea with long-term consequences, such as malnutrition, growth, and cognitive deficits in young children in resource-limited settings ^2– 7^. Despite the global burden of cryptosporidiosis, treatment options are limited to supportive therapy and a single US Food and Drug Administration-approved drug, nitazoxanide, which has limited efficacy in malnourished children and is ineffective in immunocompromised individuals ^8– 11^. Thus, there is an urgent need for the development of novel strategies to control cryptosporidiosis, particularly in susceptible populations ^4^. Progress in this field has been severely hampered by the notorious intractability of the parasite and limited tools to study it ^4^. Recently, however, there have been some exciting technological breakthroughs which have spurred renewed efforts to understand *Cryptosporidium* at the molecular and cellular levels and the pathogenesis of cryptosporidiosis and to develop new approaches to combat this disease. This article reviews these breakthroughs and discusses the continuing challenges associated with *Cryptosporidium* research.

## Novel cell culture models enable propagation of *Cryptosporidium parvum* *in vitro*

There are several constraints to drug development for cryptosporidiosis ^4, 12^. The pathogenesis of the disease and the molecules and pathways that can be targeted for drug development are poorly understood ^13^. One of the reasons for this is the lack of primary intestinal epithelial cell (IEC) models that recapitulate normal human IEC structure and function and support robust infection and the completion of the life cycle of the parasite, permitting continuous propagation *in vitro*. Most models of *C. parvum* infection *in vitro* employ transformed or immortalized adenocarcinoma-derived human IEC lines, such as Caco-2, HCT-8, and HT29 ^14, 15^. Primary human and bovine IEC models permit infections and *Cryptosporidium hominis* and *C. parvum*, respectively ^16, 17^, and *C. parvum* infection in a non-cancer-derived IEC line, FHs 74 Int, has also been reported ^18^. However, these cell lines support *C. parvum* infection for only a few days, do not permit completion of the life cycle or continuous propagation ^12^, and can display variation in gene expression depending on culture conditions ^19^. Cell-free systems for *C. parvum* culture have been reported ^20^, but although the parasite was observed to complete its life cycle by transmission electron microscopy ^21^, these *in vitro* axenic systems do not permit investigations of the parasite interactions with the host epithelia.

Recently, however, promising cell culture models that overcome some of the limitations of earlier models have been developed. For instance, Morada *et al*. ^22^ described a culture system in a simulated gut-like environment using HCT-8 cells and polysulfone hollow-fiber technology for continuous and long-term production of oocysts. Through the use of specialized equipment and culture medium supplements, *C. parvum* oocysts could be continuously propagated for more than 6 months. Various developmental stages were identified by scanning electron microscopy of infected host cells after 8 weeks of parasitic growth and by antibody staining using anti-sporozoite and anti-oocyst wall antibodies SPORO-GLO and CRYPT-A-GLO ( http://waterborneinc.com/crypt-a-glo/), respectively. This system promises to be useful for large-scale generation of infective oocysts. Other potential advantages include the availability of a large surface area for efficient growth of host cells at high cell densities comparable to *in vivo* conditions and the creation of a biphasic medium that mimics the anaerobic gut environment. However, this technology is not readily scalable and is not practical for the screening of drugs or for investigations of pathogenesis in multiple replicates in real time. In addition, the hollow-fiber system employs the transformed HCT-8 cell line that may not replicate the structure and function of primary human intestinal cells.

Miller *et al*. ^23^ developed a culture system for the propagation of infective *C. parvum* oocysts employing the human esophageal squamous cell carcinoma cell line COLO-680N. Infection with two different *C. parvum* strains resulted in the production of sufficient amounts of infective oocysts (identified by CRYPT-A-GLO) to enable continuous propagation of the parasite. Lipidomics and atomic force microscopy imaging confirmed the presence of oocysts in the culture. This seems to be a promising model for propagation of oocysts, but it uses an esophageal squamous carcinoma-derived line rather than primary human IECs, the natural habitat of anthroponotic *Cryptosporidium* spp.

**Figure 1.**
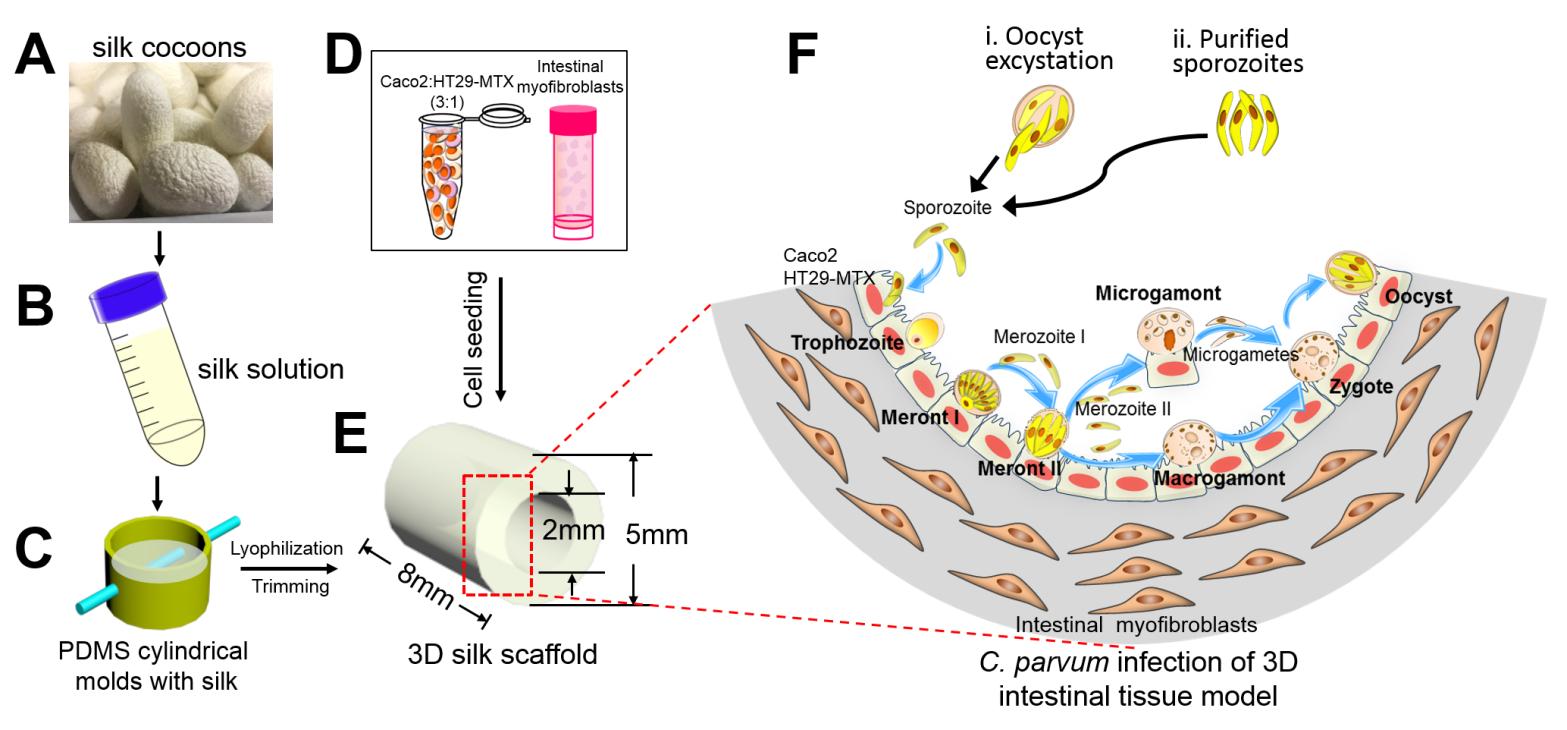
Bioengineered three-dimensional human intestinal tissue model. Silk cocoons ( **A**) were processed to yield a viscous silk solution ( **B**). ( **C**) The silk solution was poured into cylindrical molds, and a wire was inserted to develop a lumen equivalent. Caco-2 and HT29-MTX cells ( **D**) were seeded into the lumen ( **E**), while the porous bulk space was seeded with H-InMyoFibs. ( **F**) The Caco-2 and HT29-MTX cells in the lumen were infected with *Cryptosporidium parvum* oocysts or purified sporozoites. Intracellular development through asexual and sexual cycles occurred to complete the life cycle with the formation of oocysts. 3D, three-dimensional; PDMS, polydimethylsiloxane. Figure reproduced with permission from the American Society for Microbiology ^23^.

Two-dimensional culture systems are not ideal, since they do not represent the three-dimensional (3D) topology of the intestine *in vivo*. DeCicco Repass *et al*. ^24^ used a novel 3D bioengineered human intestinal tissue system for *C. parvum* infection. This model employs a porous silk protein scaffolding system with a lumen that is seeded with human IEC lines Caco-2/HT29-MTX and a “bulk” space surrounding the lumen that is seeded with human myofibroblasts (H-InMyoFibs) which secrete growth factors that support IEC growth ^25^ ( Figure 1). Immunofluorescence staining using monoclonal antibody (mAb) 4E9 which recognizes a glycopeptide epitope ^26^ and confocal microscopy was used to identify invasive and intracellular *C. parvum* stages. Infection in this model lasted for at least 17 days (the longest time tested). Importantly, contents from infected scaffolds could be transferred to fresh scaffolds to establish new infections for at least three rounds, suggesting that infection might be propagated for a longer term. Completion of the life cycle, with the formation of new oocysts identified by CRYPT-A-GLO, occurred in this model, which can be used to evaluate pathogenic processes and appears to be amenable to rapid drug screening. However, the small size of the model is a limitation for large-scale propagation. Additionally, the culture system employs transformed adenocarcinoma-derived Caco-2 and HT29-MTX cells, which are not representative of primary human IEC cells *in vivo* ^12^.

This issue was addressed using primary human intestinal enteroid cells to replace the transformed IEC lines in the system ^27^. Enteroids are stem cell-derived 3D structures that can be generated from crypts derived from human intestinal biopsies, can be passaged indefinitely, and are a more physiological alternative to transformed cell lines ^28, 29^. This 3D *ex vivo* model supports robust *C. parvum* infection and results in the production of oocysts ^30^. The use of murine-derived enteroids for *C. parvum* infection has also been reported ^31^. Recently, Heo *et al*. used human lung and intestinal epithelial organoids and CRYPT-A-GLO and SPRO-GLO antibodies to demonstrate that these systems can support the complete life cycle of *C. parvum* ^32^. Although the newly generated oocysts were infectious *in vivo*, the yield and efficiency of infection were low, and compared with the hollow-fiber system, these organoids could support continuous culture for only 28 days concurrent with a decrease in parasite number over time.

## The molecular biology toolbox expands

**Figure 2.**
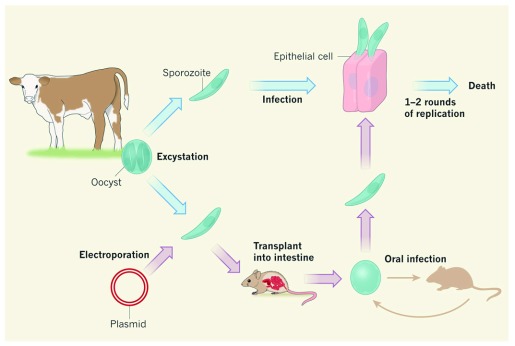
Genetic modification of *Cryptosporidium*. *Cryptosporidium parvum* oocysts isolated from the feces of infected calves can be excysted to release sporozoites which can infect mammalian epithelial cells in culture but only for one or two rounds of replication before they die. Vinayak *et al*. used CRISPR/Cas9 technology to genetically modify *C. parvum* sporozoites ^33^. Selection and replication of modified parasites requires direct injection into surgically isolated intestines of interferon-gamma knockout mice. Modified oocysts collected from mouse feces can be analyzed in culture or used to inoculate new mice to maintain the transgenic line. Figure reproduced with permission from Springer ^39^.

Until recently, reverse genetics approaches were not tractable for *Cryptosporidium*, making the elucidation of virulence factors and pathogenic pathways and the validation of drug targets next to impossible ^4, 13^. In a transformative breakthrough for the field ( Figure 2), Vinayak *et al*. ^33^ tested their optimized transfection protocol by targeting the thymidine kinase (TK) gene of *C. parvum*. Parasites were transfected with a vector encoding a guide RNA specific to TK and the *Streptococcus pyogenes* Cas9 endonuclease, which introduces a double-stranded break into the TK locus. Expression of the guide RNA and of Cas9 was driven by *C. parvum* promoters. In addition, parasites were co-transfected with a donor DNA, which serves as a template to repair the double-stranded break introduced by Cas9. This donor DNA encoded a fusion protein of the Nluc luciferase reporter gene and the Neo ^R^ resistance gene, whose expression was driven by the *C. parvum* enolase promoter. To promote homologous recombination, the donor DNA included flanking sequences complementary to regions upstream and downstream of the endogenous TK gene. This work resulted in the successful creation of a stable *C. parvum* TK knockout line ^33^.

Several components of the canonical RNA interference (RNAi) pathway are not encoded in *Cryptosporidium* genomes, precluding RNAi mechanisms for gene silencing ^34, 35^. In this pathway, double-stranded RNA (dsRNA) with sequence complementarity to an mRNA of interest is processed into single-stranded RNA (ssRNA), leading to the cleavage of the target mRNA by the enzyme Argonaute 2 (Ago2). Castellanos-Gonzalez *et al*. ^36^ addressed this limitation by transfecting parasites with human Ago2 protein, which was preloaded with ssRNAs against *C. parvum* transcripts, resulting in the reduced expression of targeted transcripts.

Another molecular mechanism of gene silencing was developed for *C. parvum* through the use of morpholinos ^37, 38^. Morpholinos are synthetic DNA analogs that inhibit protein translation initiation by base pairing to complementary mRNA. In a recent study, excysted sporozoites and HCT-8 cells were treated with morpholinos targeting *C. parvum* lactate dehydrogenase (CpLDH) and putative arginine n-methyltransferase (CpAMT) prior to *in vitro* infection, resulting in decreased protein expression of CpLDH and CpAMT ^37^. In a follow-up study, morpholinos optimized for *in vivo* delivery against CpLDH and Cp15/60 were injected intraperitoneally into interferon-gamma knockout mice, which then were infected with *C. parvum* oocysts. Expression of both proteins decreased following infection in morpholino-treated mice ^38^. Whether or not protein expression rebounds during the course of subsequent *in vitro* infection using these purified oocysts was not ascertained.

## The big data approach to transcriptome analysis

To elucidate parasite pathways that are important for pathogenesis, it is essential to examine the gene expression profile of *C. parvum* during infection. RNA sequencing (RNA-Seq) has been an invaluable high-throughput, deep-sequencing-based approach to mapping the full cellular transcriptome. This big data approach allows researchers to probe host–pathogen interactions by analyzing transcriptomic changes in both host and pathogen at any point in time. In a recent study ^40^, RNA from *C. parvum* infection of the porcine intestinal cell line IPEC-J2 was harvested 24 hours after infection and subjected to RNA-Seq analysis. The transcriptomes of oocysts alone and uninfected IPEC-J2 cells were also mapped as controls. This study identified genes involved in ribosome biogenesis and translation as being upregulated in *C. parvum* during infection *in vitro* compared with oocysts. In addition, cell division pathways were upregulated in infected versus uninfected IPEC-J2 cells ^40^. *C. parvum* transcripts accounted for only 2.2% of the total reads in this study, but whether that proportion was sufficient to measure low-level transcripts was not addressed. Another RNA-Seq study compared the transcriptome of *C. parvum* sporozoites purified from oocysts, the intestine of infected calves, or infected HCT-8 cells ^41^. General metabolic pathways were upregulated during *in vivo* infection compared with sporozoites alone. In addition, mucins, which have been shown to be involved in host cell attachment ^13^, were upregulated *in vivo*. Genes encoding oocyst wall proteins were upregulated during both *in vitro* and *in vivo* infection compared with sporozoites ^41^. However, this study did not assess transcriptomic changes in host cells.

## The search for novel drugs continues

**Figure 3.**
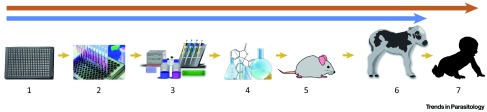
A drug-discovery screening pipeline for *Cryptosporidium*. (1) A high content imaging infection assay *in vitro* was used to identify *Cryptosporidium parvum* inhibitory compounds. (2) Secondary screening using a cytopathic effect-based assay was used to identify imidazopyrazines and pyrazolopyridines with inhibitory activity against *C. parvum* and *Cryptosporidium hominis.* (3) Identification, expression, and enzymatic activity of the *C. parvum* phosphatidylinositol-4-OH kinase (PI4K). (4) Pharmacokinetics and toxicity testing of KDU731. (5) Activity of KDU731 against transgenic *C. parvum* infection in interferon-gamma knockout mice. (6) Activity of KDU731 against native *C. parvum* infection in the neonatal calf model. (7) Additional preclinical evaluation is needed before initiation of human clinical trials. Figure reproduced with permission from Elsevier ^42^.

Development of CRISPR/Cas9-based technology for genetic modification of *C. parvum* has ** paved the way for evaluating promising drug candidates using transgenic parasites in animal models ^42^ ( Figure 3). In a recent report, KDU731, a pyrazolopyridine, inhibited the enzymatic activity of recombinant *C. parvum* phosphatidylinositol 4-kinase (PI4K), exhibited anti-cryptosporidial activity *in vitro*, and was pharmacologically non-toxic ^43^. Treatment with KDU731 of interferon-gamma knockout mice infected with transgenic *C. parvum* resulted in a marked decrease in intestinal colonization and diminished oocyst shedding ^43^. Moreover, KDU731 was efficacious in a calf model of cryptosporidiosis, leading to decreased oocyst shedding and a reduction in diarrhea. However, treatment did not entirely eliminate parasite shedding or diarrhea in these animals ^43^.

Bumped kinase inhibitors (BKIs) are a series of compounds that inhibit calcium-dependent protein kinase 1 (CDPK1) activity in apicomplexan parasites. Several studies to determine the efficacy and safety of BKI in the treatment of cryptosporidiosis have been published recently ^44– 47^. Several of these compounds have been found to reduce the severity of diarrhea and fecal oocyst shedding in calf and piglet models of cryptosporidiosis caused by *C. parvum* and *C. hominis* ^44, 45, 47^. Unfortunately, the compounds tested were not fully curative, and many displayed cytotoxicity, cardiotoxicity, or fetal toxicity (or a combination of these) in a variety of safety tests ^44, 45, 47^.

Recently, MMV665917, from the Medicines for Malaria Venture “Malaria Box” of drugs ^48^, was found to be efficacious against cryptosporidiosis in immunocompromised mice and neonatal calf models ^49, 50^. The severity of diarrhea and oocyst shedding in the calf model was reduced but not eliminated ^50^. The mechanism of action of MMV665917 remains unknown, and toxicity assessments must be performed to establish safety in humans.

An alternative approach to drug discovery is to screen for inhibition of a specific parasite target. A screen of the Prestwick Chemical Library for compounds that inhibit recombinant *C. parvum* glucose-6-phosphate isomerase (GPI) activity identified ebselen, a selenium-containing aromatic compound, which inhibited *C. parvum*, but not human, recombinant GPI activity ^51^. Likewise, ebselen inhibited *C. parvum* growth during *in vitro* infection ^51^. However, the selectivity index—the ratio of 50% HCT-8 toxicity to 50% parasite inhibition—was only about fourfold, and efficacy in animal models was not ascertained ^51^.

## Ongoing limitations

Despite these recent advances, a number of limitations persist and should be addressed in future studies. For example, none of the newer cell culture systems is ideal in all respects. Many of these systems have limited visualization of parasite growth and development. Most models employ antibody-based detection by immunofluorescence. Until recently, specific antibodies to most developmental stages were not available. Wilke *et al*. recently reported development of *C. parvum* stage-specific murine mAbs ^52^ which will be useful for identifying the intracellular stages present in the various models. Similarly, scanning or transmission electron microscopy relies on morphological assessment of which stages can be visualized. With the current availability of genetic manipulation technology for *Cryptosporidium*, epitope tagging with fluorescent tags or tags which can be fluorescently labeled with specific antibodies can be used to assess the presence of various stages.

The 3D bioengineered model, employing primary human enteroid cells, is one of the most exciting approaches but could be enhanced by incorporating additional cell types (such as nerve, immune, and endothelial cells), co-culturing with gut microbiota, and introducing physiological conditions (such as flow, peristalsis, and low oxygen tension) to develop an integrated “mini-gut” system.

Generating transgenic *C. parvum* strains using CRISPR/Cas9-based technology will likely advance and transform research on this important parasite ^33, 39, 53^. However, the selection and maintenance of transgenic strains are currently laborious and expensive. Transgenic *C. parvum* strains cannot be propagated continually *in vitro*, requiring direct injection of transfected sporozoites into the surgically exposed small intestine of paromomycin-treated, interferon-gamma knockout mice for selection and propagation ^33^. Importantly, if a target gene is essential for the invasion of small intestinal cells, it is currently not possible to generate stable transgenic strains of these parasites. Strategies for generating conditional knockouts or for complementing deleted genes have not yet been reported. Neo ^R^, conferring resistance against paromomycin, is the only selectable marker currently available. Of the newer techniques for genetic manipulation of *C. parvum*, only the CRISPR/Cas9-based gene deletion has been reproduced in another system ^43^, and this remains the only technique for targeted deletion, as opposed to knockdown of *C. parvum* genes. Finally, genetic manipulation of *C. hominis*, the species which is most relevant for human infection, is not yet possible.

For RNA-Seq analysis to be of optimal value, a high enough proportion of host cells must be infected in order for transcriptomic changes in the host to be detected. Likewise, in order to accurately quantify changes in the transcriptome of the parasite, the proportion of parasite transcripts to host transcripts must constitute a threshold of the total RNA pool to be analyzed. These issues might be overcome by applying single-cell RNA-Seq and increasing sequencing depth (or enriching for parasite transcripts prior to sequencing or both). In such an approach, RNA-Seq-based investigations should allow scientists to explore unknown genes or pathways that are essential for survival of the parasite and thus represent potential drug targets.

Significant advances have been made in the search for novel drugs for the treatment of cryptosporidiosis. However, some of these candidates do not eliminate oocyst shedding, lead to only modest reduction in diarrhea severity or duration (or both), or have been neither rigorously tested for toxicity nor evaluated in pre-clinical trials. Nonetheless, many of these compounds are modifiable, and it might be possible to develop new analogs with enhanced efficacy and decreased toxicity to human cells. To the best of our knowledge, none of the drugs has reached the human clinical trial stage. In an effort to identify additional novel targets or inhibitors (or both), further studies using transgenic, transcriptomic, and drug screening tools are warranted.

## Summary

Here, we have reviewed recent, promising advances in novel cell culture systems, genetic and molecular techniques, and drug discovery for *Cryptosporidium*. Space constraints preclude an appraisal of other recent advances in the clinical, diagnostic, and epidemiological aspects of cryptosporidiosis, the biochemistry and cellular biology of the parasite, and the innate and adaptive immune responses to infection. However, the present review shows that recent innovations are advancing knowledge of *Cryptosporidium* and provide a basis for the development of effective and practical strategies for the prevention and control of cryptosporidiosis in the vulnerable populations who need them most.
